# The Diaphragm Muscle Manual Evaluation Scale

**DOI:** 10.7759/cureus.4569

**Published:** 2019-04-30

**Authors:** Bruno Bordoni, Bruno Morabito

**Affiliations:** 1 Cardiology, Foundation Don Carlo Gnocchi, Milan, ITA; 2 Osteopathy, School of Osteopathic Centre for Research and Studies, Milan, ITA

**Keywords:** diaphragm, fascia, myofascial, phrenic nerve, surgery, manual evaluation

## Abstract

This is a technical report describing how to use the Manual Evaluation Diaphragm (MED) scale, the only evaluation scale in the world to generate a value for the mobility of the main respiratory muscle. In a previously published paper, we described how the areas of the diaphragm should be palpated correctly because a valid manual diaphragmatic evaluation was lacking in the literature. The MED scale emerged as a logical consequence of manual palpation, to provide reference values and allow comparisons between the assessments of different health professionals in multidisciplinary teams. The scale is the first non-instrumental approach to obtaining data on diaphragm function and provides parameters by which the effects that a therapeutic approach has on the diaphragm can be measured.

## Introduction

This article describes how to use the Manual Evaluation Diaphragm (MED) scale, using two examples of clinical evaluations conducted in our cardio-respiratory department. Specifically, we describe a cardiac surgery patient suffering from chronic obstructive pulmonary disease and a patient with a left ventricular assist device (L-VAD). During cardiac surgery involving sternotomy, a phrenic nerve injury may occur for various reasons including: hypothermia, the effects of sternal retractors (mechanical trauma), decreased vascular supply to the phrenic nerve from the internal mammary artery (ischemia), abnormal position of the phrenic nerve behind the pericardium, and previous presence of adherence in the phrenic nerve path (elongated) [1-2]. Phrenic nerve injury can also occur as a consequence of ablation due to atrial fibrillation [3]. Nerve injury generally results in transient paralysis (although in a small percentage of patients, the condition is permanent), with an elevation of one side, generating limited respiratory muscle movement [1]. Decreased diaphragmatic excursion after surgery can even last for more than 12 months [4]. Patients with chronic obstructive pulmonary disease (COPD) suffer from a chronic alteration of diaphragmatic function. The limitation of progressive airflow in patients with this condition leads to pathological adaptation of the respiratory muscle, in which the lower diaphragm forms a dome in the inspiratory position. Muscle thickness is increased, particularly on the left side, with decreased mechanical excursion, likely caused by a pathological shortening of the contractile fibres. Electrical conduction of the phrenic nerve is altered, probably due to the stretching caused by the chronic lowering of the diaphragm, leading to a neuropathic syndrome [5-6]. The only manual evaluation of the diaphragm muscle, the MED scale, is useful for rapidly obtaining information on contractile capacity and focusing the clinician's attention on precise areas of the diaphragm muscle. It is also useful as a tool for comparing assessments by different operators. This scale is used in the clinic and evolved in response to the realization that it is the only manual evaluation scale for diaphragmatic mobility reported in the literature [7]. Manual evaluation of the diaphragm is an important tool for patient clinical assessment, similar to orthopaedic or neurological tests. The evaluation allows identification of areas of the diaphragm with less excursion, as well as rigid and less mobile areas, both passively and actively. Further, if the tissue has lost elasticity, palpation can reveal the presence of hypomobility [7]. The aims of this report are to make the evaluation scale more familiar and to stimulate further and broader studies to validate this evaluation scale.

## Technical report

**Figure 1:**
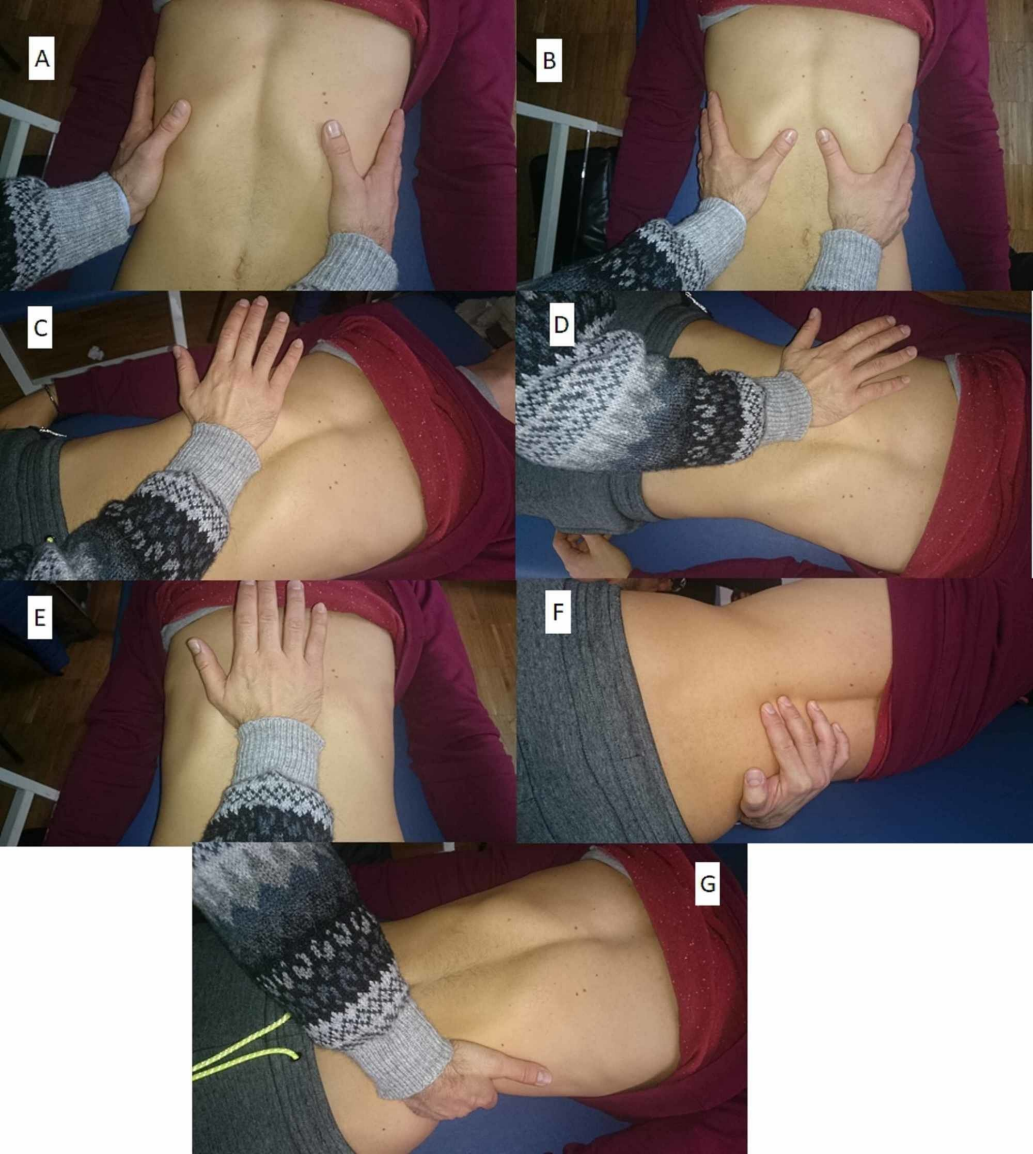
Manual evaluation of the diaphragm The operator evaluates the expansion and external rotation of the ribs during inhalation, placing their hands on the sides of the ribs (A); the anterior costal angle is evaluated during inhalation, to palpate the descent of the diaphragm muscle (B); to evaluate the diaphragmatic domes, the eminence of the hand is placed under the rib ridge, with the forearm parallel to the patient’s abdomen, and small pushes are administered with the cranial/oblique hand, in the direction of the dome, then the process is repeated from the other side (C); the posterior-lateral diaphragm mass is evaluated in the same position, but with the forearm at approximately 45 degrees from the patient’s abdomen and small stresses applied in the oblique/posterior direction (D); evaluation the xiphoid/costal area is important as the angle between the ribs and the xiphoid process opens slightly during inhalation, allowing the diaphragm to go down and come forward; to asess this, the base of the hand is placed under this area and upward stresses applied (E); the medial pillars are assessed by placing the fingers between the spinous processes of the vertebrae (between T11 and L4) and applying pressure towards the ceiling, with the patient always supine (F); the lateral pillars are evaluated by pulling the last rib to verify its elasticity (G).

Figure 1 shows the patient supine, with the operator demonstrating the use of the MED scale.

**Figure 2:**
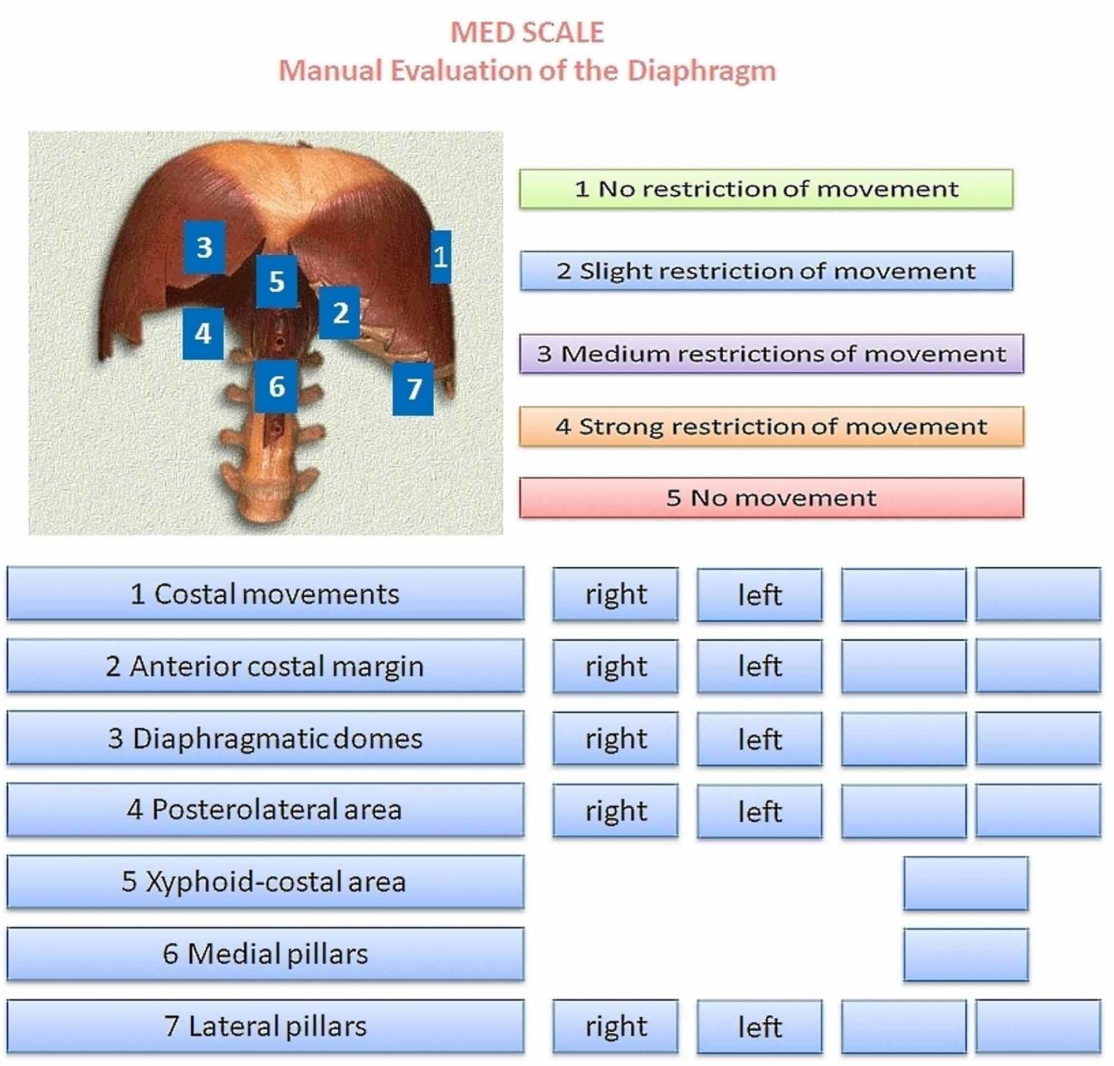
The Manual Evaluation Diaphragm (MED) scale

The MED scale is presented as a single sheet divided into four portions. In the top left of the sheet is an image of the diaphragm, including seven numbers representing the diaphragmatic portions to be evaluated by palpation, corresponding to the evaluation described above. These seven areas are shown in the bottom left of the sheet and represent both the anatomical zones of the respiratory muscle and the hierarchical course of the manual evaluation. The magnitude of the diaphragmatic dysfunction is reported in descending order by assigning a number from one to five, where one represents the absence of dysfunction and five is equivalent to a total absence of movement (active or passive). The lower right part of the scale represents the areas in which to mark values representing the mobility felt on palpation, depending on the previously published manual evaluation [7]. The use of the evaluation scale requires a little training for palpation of the diaphragm according to our already published indications. The amount of time needed to learn is very little. When the clinician has learned how to palpate the diaphragm, the use of the scale takes about a minute. The scale is a fundamental tool to identify specific areas of dysfunction. It is not possible to further speed up the palpatory evaluation, for example, palpate the movement of the diaphragm muscle in general, because the muscle is asymmetrical and moves its portions differently. A first pair of columns are highlighted (right and left) and a second pair of columns, further to the right, are completely empty. In the latter, the values detected by palpation will be marked, for the corresponding sides (right and left). The two single columns on the right represent the evaluation of the costal/xiphoid area and the medial pillars (Figure 2).

**Figure 3:**
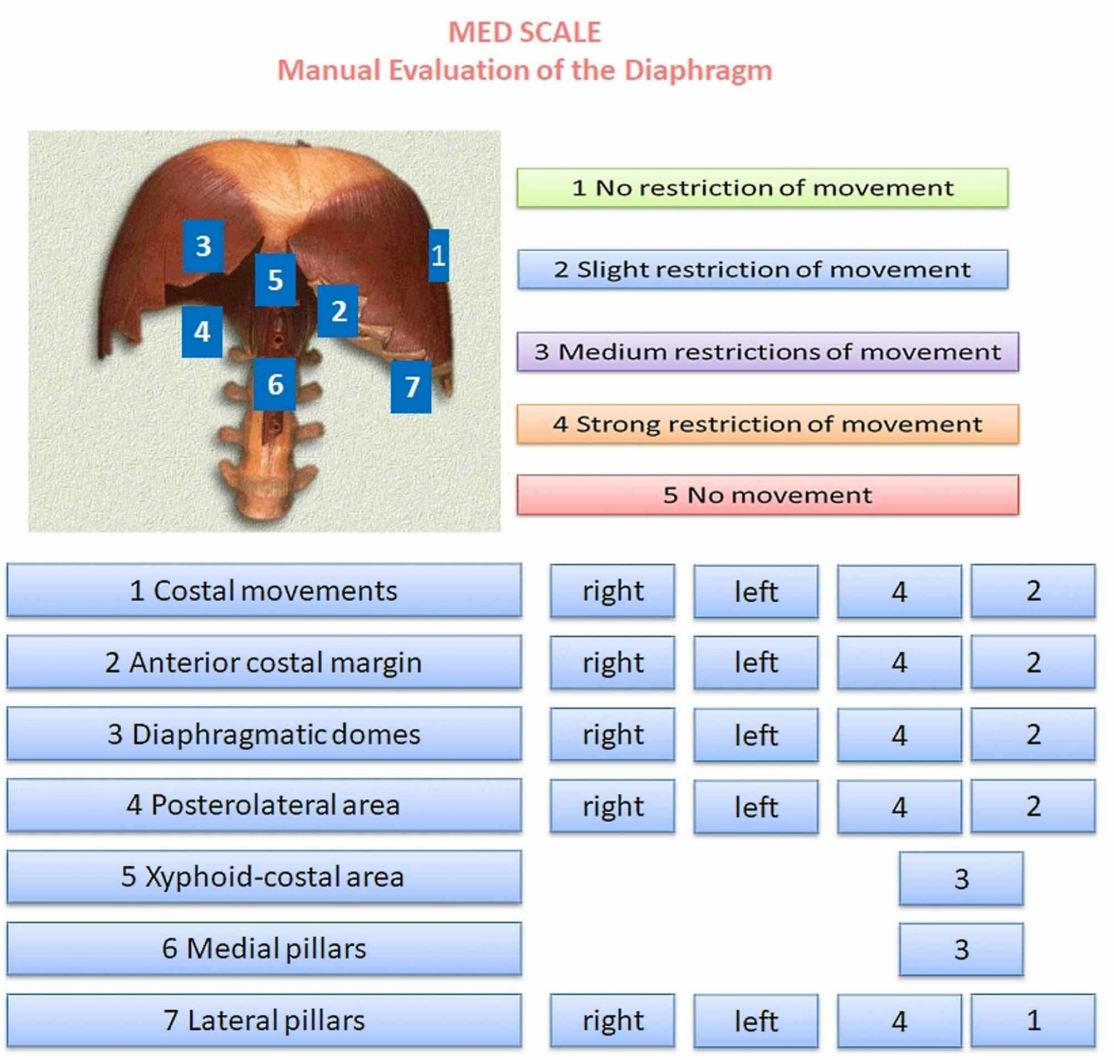
First clinical example.

Below, we describe some clinical examples of the use of the MED scale. A patient with myocardial infarction underwent angioplasty and stent placement in the obtuse marginal branch following a previous bypass and had previously also undergone mitral valve replacement and had a pacemaker fitted. The associated disease was COPD of unspecified severity. The patient presented with slight dyspnea during speech and auscultation revealed a reduced vesicular murmur across the whole area. The patient was subjected to a palpatory examination of the diaphragm using the MED scale, which revealed serious dysfunction of the right side of the diaphragmatic area, as highlighted on the MED scale presented in Figure 3. 

**Figure 4:**
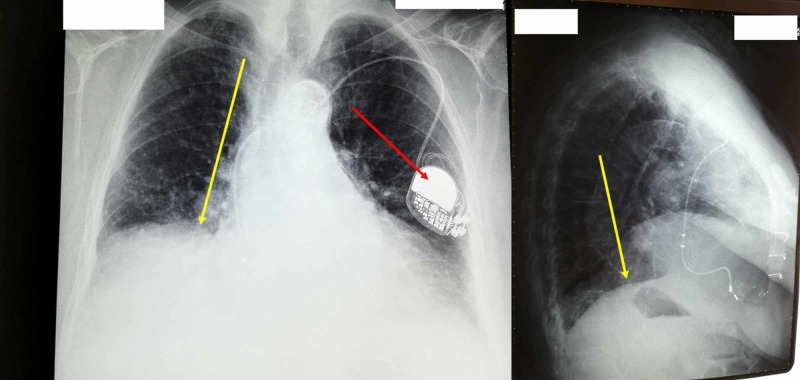
X-ray: instrumental examination showing an elevation of the diaphragm on the right side The red arrow indicates the pacemaker, the yellow arrow indicates the elevation of the right diaphragmatic dome.

When the patient was subjected to x-ray, instrumental examination showed an elevation of the diaphragm on the right side, with obliteration of the corresponding costophrenic angles and obstructed areas at the bases of the lungs (Figure 4).

**Figure 5:**
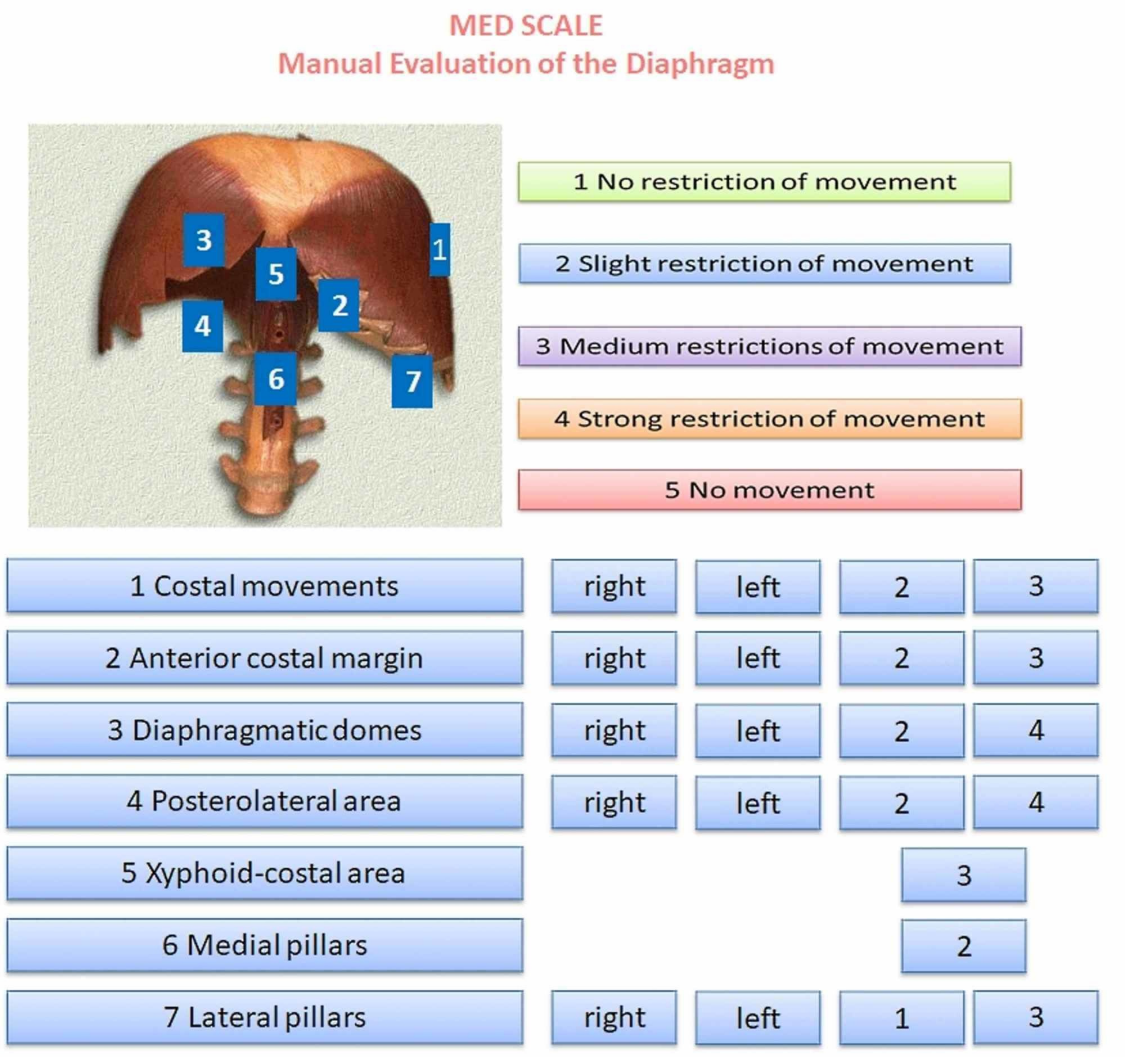
The second clinical example

A second clinical example concerns a patient who underwent insertion of an L-VAD, a mechanical pump that performs the function of the left ventricle to maintain a constant flow. This type of surgery is performed for one of two main reasons: 1) because the patient is waiting for a transplant or “bridge therapy”, or 2) to prolong the life of the patient as far as possible, known as “destination therapy” [8-9]. The patient was fitted with a Heartmate III L-VAD. On admission to the clinic, one month after the cardiac surgery, the patient did not show dyspnea during speech and on auscultation; the vesicular murmur was found to be limited to the bases. Manual evaluation by palpation of the patient’s diaphragmatic area and reporting the results on the MED scale revealed dysfunction of the left side (Figure 5).

**Figure 6:**
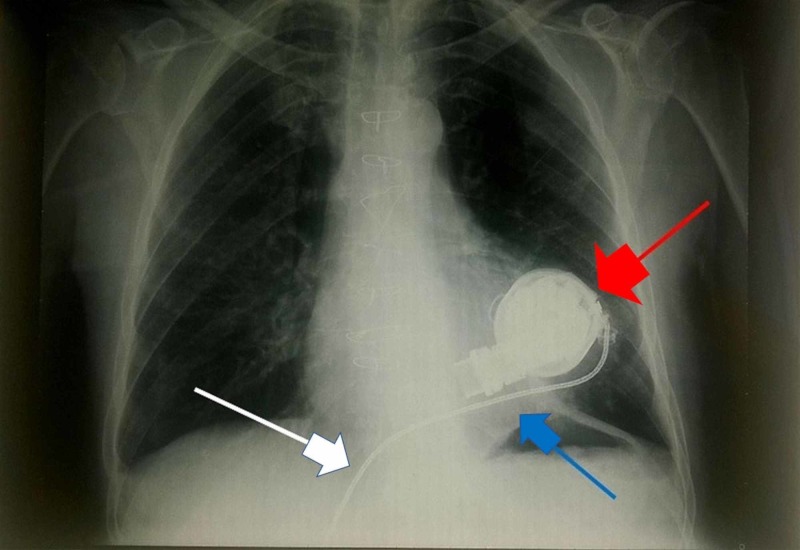
X-ray showing the position of the L-VAD The red arrow indicates the mechanical pump, the blue arrow indicates the left ventricle, and the white arrow highlights the driveline (an electric wire that connects the batteries to the pump).

The x-ray image shows the position of the L-VAD, where the red arrow indicates the mechanical pump, the blue arrow indicates the left ventricle, and the white arrow highlights the driveline (an electric wire that connects the batteries to the pump) (Figure 6).

## Discussion

The MED scale is easy to use and enhances the information gained by palpation of the diaphragm muscle. It is an effective communication tool for use by different health professionals, similar to orthopaedic or neurological tests, and is non-invasive, inexpensive, simple to learn, and not detrimental to the patient. In clinical practice, the first evaluation tools of the clinician or manual operator are orthopaedic and neurological tests. In the continuation of the evaluation, the doctor will decide which instrumental tests to perform if the first manual tests have indicated a positivity or have put the doctor in doubt. Manual evaluations are an added value to understand the symptom picture and sometimes to avoid performing further instrumental examinations. Instrumental examinations are not always able to give precise information if the cause of the symptom is subclinical, although the patient shows a symptom. The scale can be useful in the decision-making process of the clinician because palpation already indicates an area of dysfunction. By combining the MED scale result and the instrumental exam, the doctor can decide on a specific therapy (physiotherapy, pharmacology, surgery). The first evaluation described in the technical report section revealed an important dysfunction of the right side of the diaphragm muscle, later confirmed by x-ray examination. In the second clinical case, there was no clear lesion of the diaphragm; yet, the MED scale highlighted a respiratory problem on the left side. In the second patient, in addition to the weakness of the diaphragm, internal fibrous adhesions may be present in the pericardium, between the driveline and the diaphragm [10]. In patients with respiratory problems, the spirometry is a routine exam, but the forced expiratory volume in 1 second (FEV1) does not necessarily indicate the precise function of the diaphragm muscle or the presence/absence of dyspnea [11]. The availability of a concrete instrument that can assess the contractile capacity of the diaphragm is useful. Knowledge of the areas with the greatest dysfunction in diaphragm mobility provides the clinician with a comparable parameter at the end of multidisciplinary treatments. When the patient has finished the treatment previously decided by the clinician, the doctor should palpatorially re-evaluate the diaphragm and compile the MED scale with the new data. In this way, added clinical data is obtained, giving value to the therapeutic process carried out by the same patient. This evaluation scale is indicated not only for cardiology or respiratory patients, but also for patients in other areas of healthcare where it is necessary to understand how the diaphragm muscle behaves. The scale could also be usefully applied in sports, where increased respiratory performance and constant evaluation are required and where multidisciplinary teams are always involved. The next step will be the validation of the MED scale with a greater number of patients and the definition of inter-operator variability.

## Conclusions

This technical report describes how to use the MED scale, which is applicable to all areas of health and likely also in the sphere of sports, to generate a score representing the mobility of the diaphragm. This article described the use of the scale in two clinical scenarios, including a cardiology/pneumology patient with a clear lesion of the diaphragm on the right side and a patient who had undergone L-VAD implantation.
